# Human PrimPol mutation associated with high myopia has a DNA replication defect

**DOI:** 10.1093/nar/gku879

**Published:** 2014-09-27

**Authors:** Benjamin A. Keen, Laura J. Bailey, Stanislaw K. Jozwiakowski, Aidan J. Doherty

**Affiliations:** Genome Damage and Stability Centre, School of Life Sciences, University of Sussex, Brighton BN1 9RQ, UK

## Abstract

PrimPol is a primase-polymerase found in humans, and other eukaryotes, involved in bypassing lesions encountered during DNA replication. PrimPol employs both translesion synthesis and repriming mechanisms to facilitate lesion bypass by the replisome. PrimPol has been reported to be a potential susceptibility gene associated with the development of myopia. Mutation of tyrosine 89 to aspartic acid (PrimPol^Y89D^) has been identified in a number of cases of high myopia, implicating it in the aetiology of this disorder. Here, we examined whether this mutation resulted in any changes in the molecular and cellular activities associated with human PrimPol. We show that PrimPol^Y89D^ has a striking decrease in primase and polymerase activities. The hydrophobic ring of tyrosine is important for retaining wild-type extension activity. We also demonstrate that the decreased activity of PrimPol^Y89D^ is associated with reduced affinities for DNA and nucleotides, resulting in diminished catalytic efficiency. Although the structure and stability of PrimPol^Y89D^ is altered, its fidelity remains unchanged. This mutation also reduces cell viability after DNA damage and significantly slows replication fork rates *in vivo*. Together, these findings establish that the major DNA replication defect associated with this PrimPol mutant is likely to contribute to the onset of high myopia.

## INTRODUCTION

Cells are required to replicate their genomes in a faithful way to avoid mutagenesis thus maintaining genetic stability. However, DNA is highly prone to damaging agents, including oxygen-free radicals, ultraviolet (UV) light, ionizing radiation and desiccation, resulting in the production of lesions and breaks that disrupt the structure of the double helix. One of the many undesirable consequences of such damage is the disruption of the progression of DNA replicases (1), which copy the genetic material. These replication machines are exquisitely sensitive to the conformation of the template and are prone to stalling upon encountering DNA damage. To overcome such genetic obstacles, cells have evolved a variety of lesion tolerance pathways to allow bypass of damage and resumption of replication (2,3).

PrimPol is a recently characterized primase-polymerase, belonging to archaeo-eukaryotic primase (AEP) superfamily, that is involved in lesion bypass during both nuclear and mitochondrial replication in eukaryotic cells (4–6). PrimPol is required for the replication past particular lesions, such as UV photoproducts, that block the cellular replication machinery. It can deploy two different mechanisms, utilizing either of its polymerase and primase activities to facilitate efficient lesion bypass. PrimPol can replicate directly through a number of obstructing lesions by translesion DNA synthesis (TLS) and it can also reprime replication post-damage to facilitate fork restart (4,7,8), although the exact timing of their usage remains to be established. PrimPol knockout cells are sensitive to damaging agents (e.g. UV, 4NQO and MMS) and murine PrimPol^−/−^ cells show significant levels of DNA breaks, particularly after treatment with agents that stall replication (4).

Although recent studies have established the importance of PrimPol in damage tolerance during DNA replication in cellular and animal model systems, its association with human diseases has only been conjectured. However, exome sequencing of the genomes of candidate patients has recently implicated the PrimPol gene as a possible susceptibility gene associated with high myopia (9). This study identified a missense mutation in human PrimPol caused by a thymine to guanine transversion, resulting in the substitution of tyrosine at position 89 by an aspartic acid residue (Y89D). This mutation was not only found in a single family but also four additional sporadic patients with high myopia and these patients had high refractive errors (−6.5 to −32.15 diopters, D) (9). Myopia, or short-sightedness, is a common problem around the world in those with visual defects and this problem is rapidly increasing. Myopia accounts for 41% of decreased vision in Western populations and this up from 25% in the early 1970s (10). In some South-East Asian populations up to 80–90% of all children leaving school are now myopic (11). Notably 10–20% of these children suffer from high myopia, an extreme form of the disease, often characterized by a refractive error of less than −6.00 D and is often associated with other serious ocular disorders (11,12). The aetiology of high myopia remains extremely complex and involves the synergy between a number of genes.

**Figure 1.**
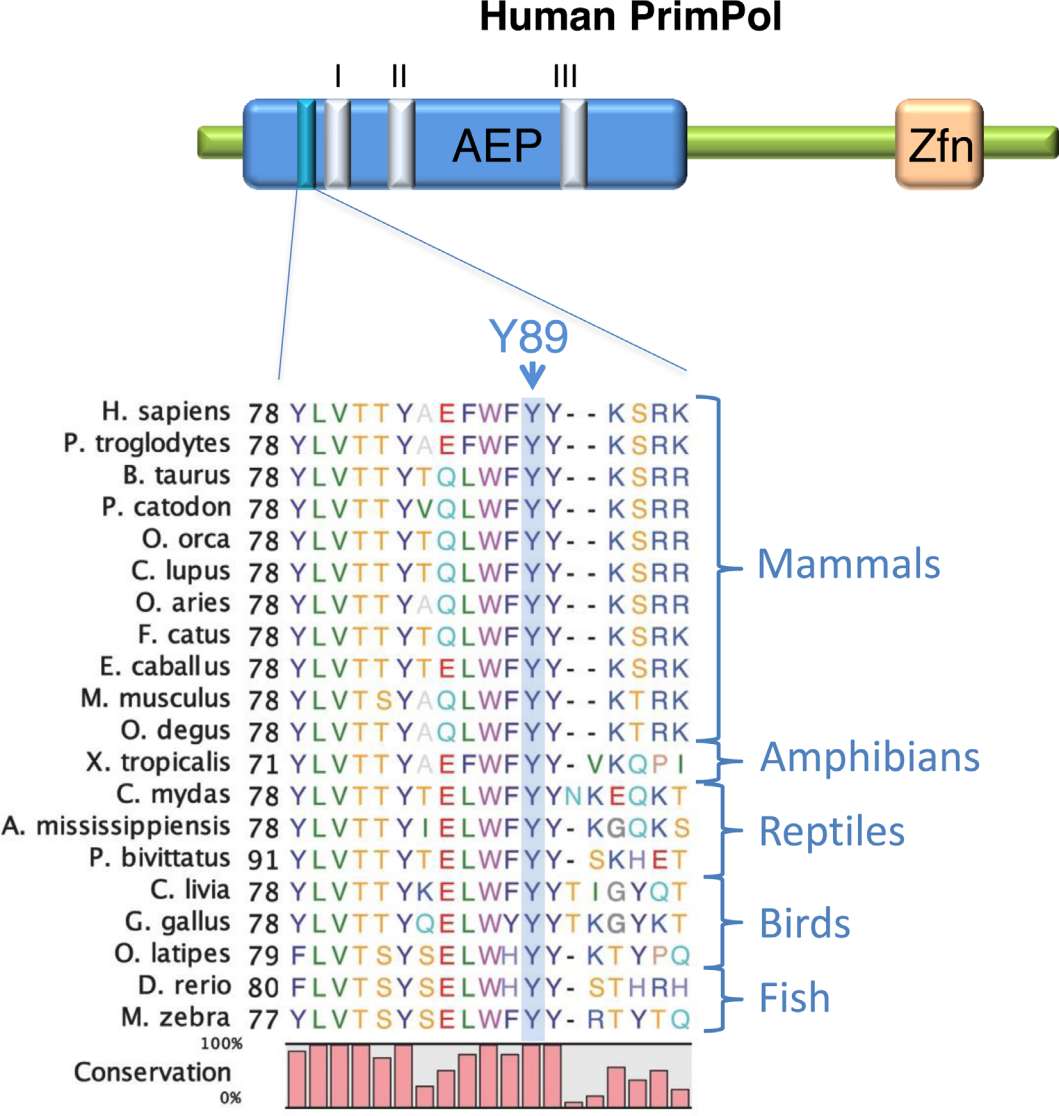
PrimPol^Y89D^ mutation is located adjacent to active site Motif I. An alignment of PrimPol protein sequences from a variety of chordates, including examples of mammals, reptiles, amphibians, birds and fish. The position of the mutated tyrosine 89 in human PrimPol is denoted by an arrow and is labelled ‘Y89’. The relative proximity of this residue to the catalytic motifs (I–III) is shown in the AEP domain. The zinc finger domain is denoted by ‘Zfn’.

The active site of PrimPol is predicted to consist of three magnesium-coordinating, negatively charged residues: an aspartate at residue 114, a glutamate at residue 116 and an aspartate at residue 280 (4). Although the tyrosine at position 89 is not predicted to reside directly within the active site, it is in close proximity and therefore may play yet undetermined roles in the activities associated with these enzymes. The mutated tyrosine (Y89) is a highly conserved residue across a wide array of chordate species (Figure 1) and *PolyPhen-2* prediction programme indicates that mutation of this residue would be functionally damaging (9). In addition to PrimPol, this tyrosine is also present in the related predicted primase PF14_0050 in *plasmodium falciparum*, so its evolutionary conservation is indicative of a significant functional residue (13).

In this study, we aimed to characterize the biochemical properties of the Y89D mutant of PrimPol (PrimPol^Y89D^) to determine if this amino acid change alters PrimPol's activities thus establishing a molecular basis for its proposed involvement in high myopia. Here, we demonstrate that the Y89D mutant loses its ability to prime using ribonucleotides (rNTPs), but can still prime using deoxyribonucleotides (dNTPs). PrimPol^Y89D^ has a greatly reduced template-dependent processivity activity, when compared with the wild-type enzyme. In addition, we report that this mutant has significantly reduced binding for both DNA and dNTPs but its fidelity remains largely unchanged. Furthermore, we show that it is the hydrophobic ring of the tyrosine at position 89 that is important for maintaining these activities. We also demonstrate that changes in enzyme activity are the result of global changes in the structure of the polymerase domain of PrimPol. Finally, we report that this mutation significantly slows replication fork rates *in vivo* and reduces cell viability after DNA damage. Together, these data establish that a point mutation identified in PrimPol from patients with high myopia results in a major disruption of the catalytic and replication activities associated with human PrimPol thus establishing a link between replication stress and human disease, particularly high myopia.

## MATERIALS AND METHODS

### Construction of human PrimPol mutants

Wild-type human PrimPol was cloned as described previously (4). PrimPol^Y89D^, PrimPol^Y89F^ and PrimPol^Y89S^ were cloned by site-directed mutagenesis using the primers that can be found in Supplementary Table S1. PrimPol_1–354_ was cloned as described previously (14) and PrimPol^Y89D^_1–354_ was cloned by site-directed mutagenesis from this construct using the primers in Supplementary Table S1.

### Expression and purification of recombinant PrimPol proteins

Wild-type PrimPol, PrimPol^Y89D^, PrimPol^Y89F^ and PrimPol^Y89S^ were expressed in BL21 (pLysS) *Escherichia coli* cells overnight at 16°C and PrimPol_1–354_ and PrimPol^Y89D^_1–354_ were expressed overnight at 25°C. The proteins were then purified as described previously (14). Protein concentrations were determined using the absorbance at 280 nm and the extinction coefficient for each of the protein constructs.

### DNA primase assays

DNA primase assays were carried out as described previously (14). The templating oligonucleotide sequences can be found as sequences 1–4 in Supplementary Table S2. Products were resolved on a 15% (v/v) polyacrylamide gel containing 7 M urea and 1× TBE buffer at 850 V for 2.5 h in 1× TBE buffer. Fluorescently labelled DNA oligomers were detected by scanning using a Fujifilm FLA-5100 image reader.

### DNA primer extension assays

Polymerase activity was determined by DNA primer extension assays. Hex-labelled fluorescent primers were annealed to unlabelled templates (sequences in Supplementary Table S2). Note that 100 nM of enzyme was incubated at 37°C with 20 nM DNA, 10 mM Bis-Tris-Propane-HCl (pH 7.0), 10 mM MgCl_2_, 1 mM DTT and 200 μM dNTPs (Roche) to a final volume of 20 μl for time points increasing up to 60 min. Reactions were quenched using 2× stop buffer (95% formamide, 0.09% xylene cyanol, 0.05% bromophenol blue, 200 nM competitor oligonucleotide) and boiled at 95°C for 5 min. Products were resolved by electrophoresis as in the DNA primase assays. For assessment of the fidelity of PrimPol_Y89D_, oligonucleotides with two adjacent, identical bases downstream of the primer were incubated, along with 100 nM enzyme, with single nucleotides for 20 min at 37°C and were subsequently resolved as above.

### Electrophoretic mobility shift assays (EMSAs)

EMSAs were carried out on an overhanging DNA substrate (sequences 5 and 6 annealed from Supplementary Table S2). Varying concentrations of PrimPol_1–354_ and PrimPol^Y89D^_1–354_ were added to 60 nM DNA, 10 mM Bis-Tris-Propane-HCl (pH 7.0), 10 mM MgCl_2_ and 1.0 mM DTT to a final volume of 20 μl and incubated at 25°C for 60 min. The reactions were supplemented with 2 μl 25% (w/v) ficoll. Samples were resolved by electrophoresis on a 5% (v/v) polyacrylamide gel containing 0.5× TBE buffer at 150 V for 0.5 h, then 300 V for 2.5 h in 0.5× TBE buffer. Fluorescently labelled DNA oligomers were detected by scanning using a Fujifilm FLA-5100 image reader.

### Single turnover kinetic assays

The single turnover assays were based on a previously described protocol (15). Hex-labelled fluorescent DNA (20 nM; sequences 6 and 11 annealed from Supplementary Table S2) was incubated with wild-type and Y89D PrimPol (200 nM). The DNA/PrimPol mixture was incubated with varying concentrations of dATP for time points ranging from 0.5 to 60 min and each reaction was carried out in triplicate. Reactions were quenched by the addition of 2× stop buffer. The products were resolved by electrophoresis as in the primase assays. The concentrations of extended and unextended fluorescent DNA primers were measured using ImageQuant software (GE Life Sciences). The concentration of extended product relative to total fluorescent DNA loaded­ was plotted as a function of time and the data were fit to a single-exponential curve to obtain the *k*_obs_ for the different dATP concentrations using the following exponential equation:

\begin{equation*} [product] = A(1 - e^{ - k_{{\rm obs}} t} ) \end{equation*}

A secondary plot of *k*_obs_ as a function of dATP concentration was fitted to the following hyperbolic equation:

\begin{equation*} k_{{\rm obs}} = \frac{{k_{{\rm pol}} [dATP]}}{{K_{d({\rm dNTP})} + [dATP]}} \end{equation*}

where *k*_pol_ is the rate of polymerization and *K_d_*_(dNTP)_ is the equilibrium dissociation constant for dATP.

### DNA extension processivity assays

The PrimPol variants were analysed for processivity as described previously (16). PrimPol (100 nM) was pre-incubated at 37°C with 60 nM substrate DNA (sequences 5 and 6 annealed in Supplementary Table S2), 10 mM Bis-Tris-Propane-HCl (pH 7.0), 10 mM MgCl_2_ and 1 mM DTT for 30 min. Reactions were initiated by adding deoxyribonucleotides and excess sonicated herring sperm DNA (1 mg/ml) as an enzyme trap. Reactions were quenched after time points of 15, 30, 60, 120 and 360 s with addition of 2× stop buffer and boiled at 95°C for 5 min. Samples were resolved by electrophoresis as described above.

### Circular dichroism (CD) spectroscopy

Samples were buffer exchanged into a buffer of 400 nM NaF, 15 mM Tris-HCl (pH 7.5) and concentrated to 3 mg/ml. The concentration of recombinant protein was determined before and after the CD scans, to ensure accurate secondary structure predictions. Samples were placed in a 0.1 mm quartz cuvette (Starna, Essex, UK) and measurements were taken using a JASCO J-715 spectropolarimeter (JASCO). The CD spectrum of the empty buffer was subtracted from that of the sample and time constant was set to 4 s with a scan rate of 50 nm/min. The bandwidth was 1 nm and the sensitivity set to standard. Scans were performed in triplicate from 320 to 180 nm with a 0.1 nm data pitch and continuous scan mode. A Peltier device was used to maintain a temperature at 20°C.

### Protein denaturation curves

The PrimPol_1–354_ and PrimPol^Y89D^_1–354_ constructs were both buffer exchanged into a buffer of 200 mM NaF, 15 mM Tris-HCl (pH7.5). Note that 45 μl of 1 μM protein solution was added to 15 μl of SYPRO Orange, resulting in a final protein concentration of 0.75 μM. A control of 45 μl was also added to 15 μl of SYPRO Orange. 20 μl of each sample was aliquoted in triplicate into a 96-well plate. Protein melting experiments were carried out using the LightCycler 480 System II (Roche). The instrument was configured with a detection format of 465 nm as the wavelength of excitation and 580 nm as the emission wavelength to detect SYPRO Orange-specific signal. Denaturation curve fluorescent signal was acquired within a range of 20°C–80°C using a ramping rate of 0.03°C s^−1^ and an acquisition of 20 data points per degree celsius. Melting temperatures (*T*_m_) were determined through the measurement of the lowest point of the negative differential of the denaturation curve. Data was corrected for the background signal of the buffer conditions.

### Complementation and survival assays in PrimPol^−/−^ avian cells

DT40 cells were grown at 39°C in RPMI 1640 medium supplemented with 10 μM β-mercaptoethanol, 1% L-glutamine, 1% penicillin streptomycin, 10% fetal calf serum, 1% chicken serum (Sigma). PrimPol^−/−^ cells were complemented with human PrimPol^Y89D^ cloned into pCI-neo as described previously (14) and expression was confirmed by western blotting alongside wild-type complemented stable cells generated previously (14). DNA replication fork speed and stalling after UV damage was measured by fibre analysis as described previously and carried out in triplicate (4). Cell survival after UV-C damage was measured using CellTiter-blue (Promega). Briefly, 40 × 10^4^ cells (or a serial dilution for decreased damage) were resuspended in a small volume of phosphate buffered saline and exposed to increasing doses of UV-C, media was replaced and cells were grown for 48 h. Cells were then transferred to 96-well plates and viability was measured with CellTiter-blue.

## RESULTS

### PrimPol^Y89D^ retains DNA primase but loses its RNA primase activity

**Figure 2.**
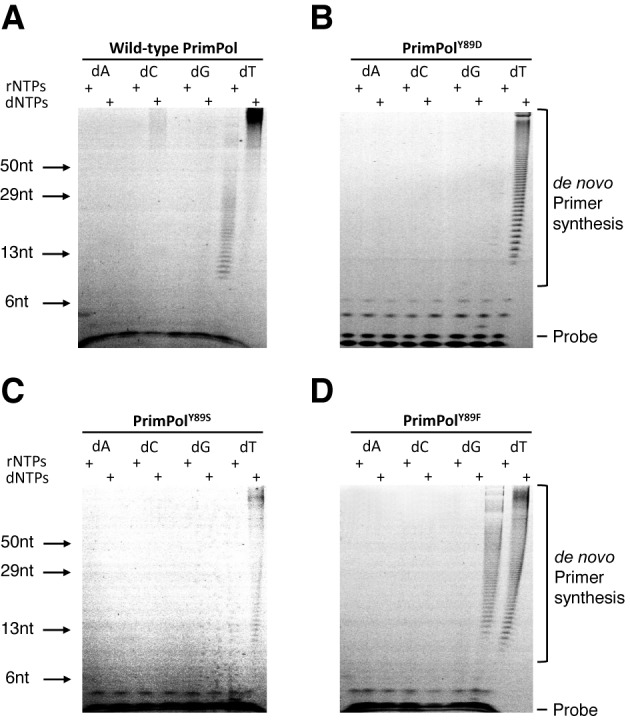
Primase activities of human PrimPol and PrimPol^Y89D^. (A) Human PrimPol has primase activity and can produce *de novo* primers using rNTPs and dNTPs in a homo-polymer template-dependent fashion. (B) PrimPol^Y89D^ partially loses its primase activity. This variant of PrimPol cannot synthesize RNA primers but is an active primase utilizing dNTPs. (C) PrimPol^Y89S^ shows significant loss of the enzymatic activity with residual primase activity observed in presence of dNTPs. (D) PrimPol^Y89F^ is an active primase variant capable of synthesizing both RNA and DNA primers with efficiency comparable to wild-type PrimPol.

To determine if PrimPol displays an underlying biochemical defect that contributes to the high myopia clinical phenotype, we first examined the primase activity of the PrimPol Y89D (PrimPol^Y89D^) variant. *De novo* primer synthesis remained largely unchanged between the mutant and the wild-type in the production of DNA primers from dNTPs (Figure 2). However, unlike the wild-type enzyme, PrimPol^Y89D^ was unable to synthesize RNA primers in the presence of even high concentrations of rNTPs. While it has been reported that PrimPol is more proficient at producing DNA over RNA primers *in vivo*, we remained intrigued as to why this mutation in PrimPol conferred a defect in RNA primer synthesis. To determine whether this disruption in activity was a result of the carboxylic acid moiety of the aspartate or was due to the removal of the hydrophobic ring of tyrosine, we produced two additional PrimPol variants, Y89S and Y89F. The serine side-chain of Y89S could discriminate whether the charge of the aspartate caused the altered activity we observed with Y89D while retaining the hydroxyl group of tyrosine, and the phenylalanine of Y89F could help determine if it was the hydrophobic ring of tyrosine that was important for retaining rNTP primase activity. Upon testing Y89S and Y89F for primase activity (Figure 2), we found that the PrimPol^Y89S^ has an activity comparable to Y89D but the Y89F variant of PrimPol returned primase activity back to wild-type, restoring the ability of the enzyme to produce RNA primers.

### PrimPol^Y89D^ mutant has limited processivity

**Figure 3.**
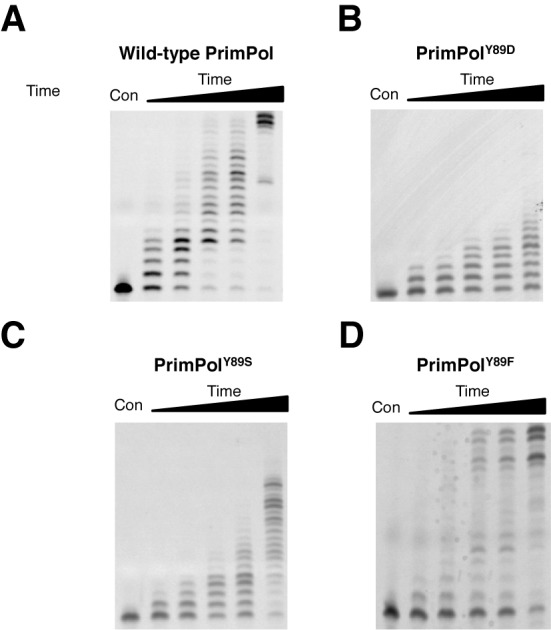
Primer extension activities of human PrimPol and PrimPol^Y89D^. (A) Human PrimPol has primer extension activity and produces full-length templates following incubation for up to 60 min (lane 1 is a control and lanes 2–6 represent time points at 0.5, 1, 3, 5 and 60 min). (B) PrimPol^Y89D^ shows a marked reduction in polymerase activity, incorporating up to eight nucleotides following incubation for 60 min. (C) PrimPol^Y89S^ also shows a loss in polymerase activity relative to wild-type PrimPol, though it is a more active polymerase than PrimPol^Y89D^. (D) PrimPol^Y89F^ restores polymerase activity. The Y89F variant can fully extend primers but there is an increased level of unextended primer relative to wild-type PrimPol.

PrimPol comprises two intrinsic catalytic activities. To determine any catalytic deficiencies associated with the Y89D point mutation, we must also consider its polymerase activity in addition to its primase activity (Figure 3). Primer extension assays on PrimPol showed that the polymerase activity of PrimPol^Y89D^ was significantly reduced in comparison to the wild-type enzyme. We next tested if this activity was restored by either Y89S or Y89F variants of PrimPol (Figure 3) and observed that Y89S had slightly higher activity than the Y89D variant but was not restored to wild-type levels. In contrast, Y89F was fully proficient at extending primers opposite a templating strand. Notably, although PrimPol^Y89F^ was able to fully extend primers, and showed full extension at time points as short as 3 min (Figure 3D, lane 4), more primers were left unextended compared with wild-type PrimPol, suggesting that this enzyme has higher activity once it has bound DNA but coordinates it less efficiently.

**Figure 4.**
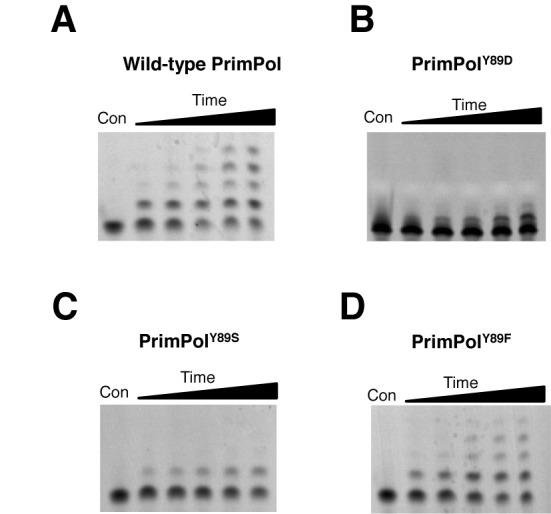
Processivity of human PrimPol and PrimPol^Y89D^. Primer extension assays were performed in the presence of an excess of herring sperm DNA trap to ensure the polymerase only binds once to the template DNA. Control reactions were also carried out to test the effectiveness of the trap DNA (data not shown). The first lanes represent a no-enzyme control and lanes 2–6 represent time points of 15, 30, 60, 120 and 360 s. (A) Human PrimPol is a poorly processive enzyme, inserting up to four nucleotides opposite a templating DNA strand in a single binding event. (B) PrimPol^Y89D^ has marked reduction in processivity, and is only able to insert a single nucleotide opposite a templating DNA strand per single binding event. (C) PrimPol^Y89S^ can also insert only one single nucleotide opposite a templating DNA strand. (D) PrimPol^Y89F^ restores processivity and has the ability to insert up to four nucleotides, similarly to wild-type PrimPol.

PrimPol was previously shown to be a distributive DNA polymerase, inserting only up to four nucleotides during a single binding event (Figure 4). We next determined whether the processivity of the mutant was altered, causing the observed reduction in the polymerase activity. PrimPol^Y89D^ variant could only insert a single nucleotide opposite a templating strand of DNA, making it an even more distributive enzyme than wild-type PrimPol (Figure 4). To determine whether the aromatic ring of tyrosine was the main determinant for enzyme activity, we tested the processivity activities of Y89S and Y89F variants. Consistent with Y89D, PrimPol^Y89S^ could only insert a single nucleotide. However, in common with the wild-type enzyme, PrimPol^Y89F^ could insert up to four nucleotides. This establishes that the presence of an aromatic side-chain at this position is requisite for maintaining more processive extension by PrimPol.

### Reduced DNA binding contributes to the low processivity of PrimPol^Y89D^

**Figure 5.**
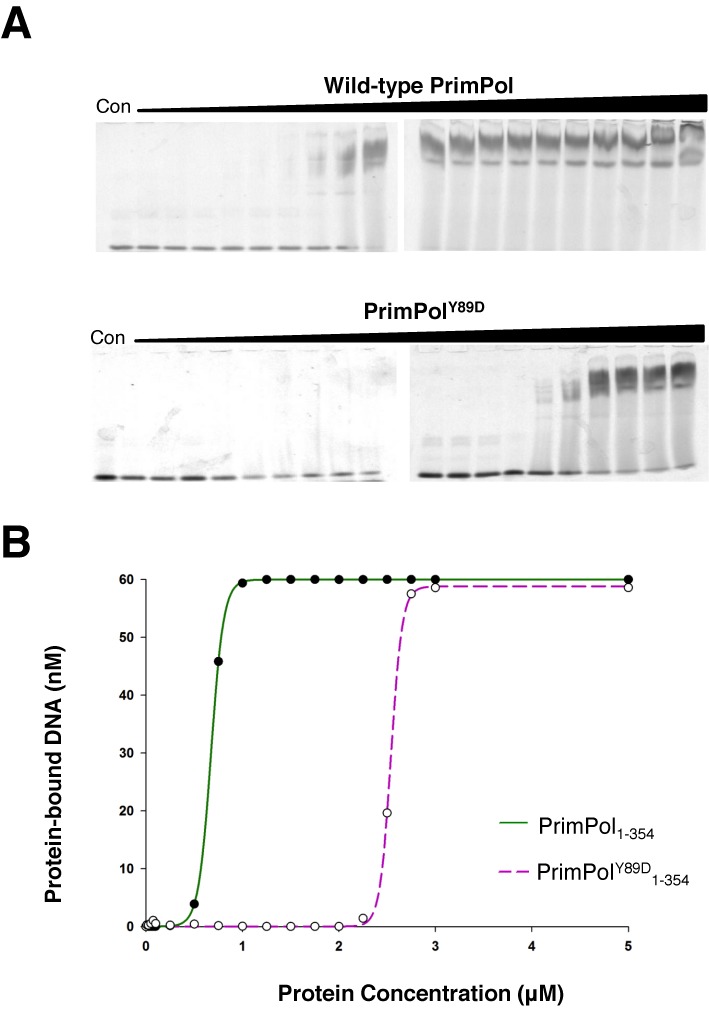
DNA binding efficiency of polymerase domains of wild-type and PrimPol^Y89D^. (A) The polymerase domains (PrimPol_1–354_) for both wild-type and PrimPol^Y89D^ were used to eliminate the effects of binding from the zinc finger domains. Wild-type PrimPol domain bound to DNA at a much lower concentration than PrimPol^Y89D^ as determined by EMSAs. The concentrations used in both of these EMSAs were 0, 0.01, 0.025, 0.05, 0.075, 0.1, 0.25, 0.5, 0.75, 1, 1.25, 1.5, 1.75, 2, 2.25, 2.5, 2.75, 3, 5 and 10 μM. (B) These assays were subsequently quantified to determine *K*_D(DNA)_ values for the polymerase domains of wild-type PrimPol (solid green line) and its Y89D variant (broken purple line). *K*_D(DNA)_ of wild-type PrimPol was 0.69 μM, while the *K*_D(DNA)_ of PrimPol^Y89D^ was 2.55 μM.

A reduction in processivity can be attributed to alterations in either dNTP or substrate DNA binding. To test the binding efficiencies of wild-type and PrimPol^Y89D^, we introduced the mutation into the polymerase domain (PrimPol_1–354_) of PrimPol. This ensured that the contributory binding effects of the zinc finger were removed to enable assessment of the alteration in binding caused solely by this point mutation in the polymerase domain. As the polymerase domain is able to bind both single- and double-stranded DNA (14), we chose to use a labelled overhanging DNA substrate in our EMSAs, similar to the substrates PrimPol would encounter *in vivo*. The binding efficiency of PrimPol_1–354_^Y89D^ to DNA was almost four times lower than PrimPol_1–354_. The wild-type *K*_D(DNA)_ was 0.67 μM, compared with Y89D *K*_D(DNA)_ of 2.55 μM (Figure 5).

### PrimPol^Y89D^ has reduced dNTP binding but its fidelity is not altered

**Figure 6.**
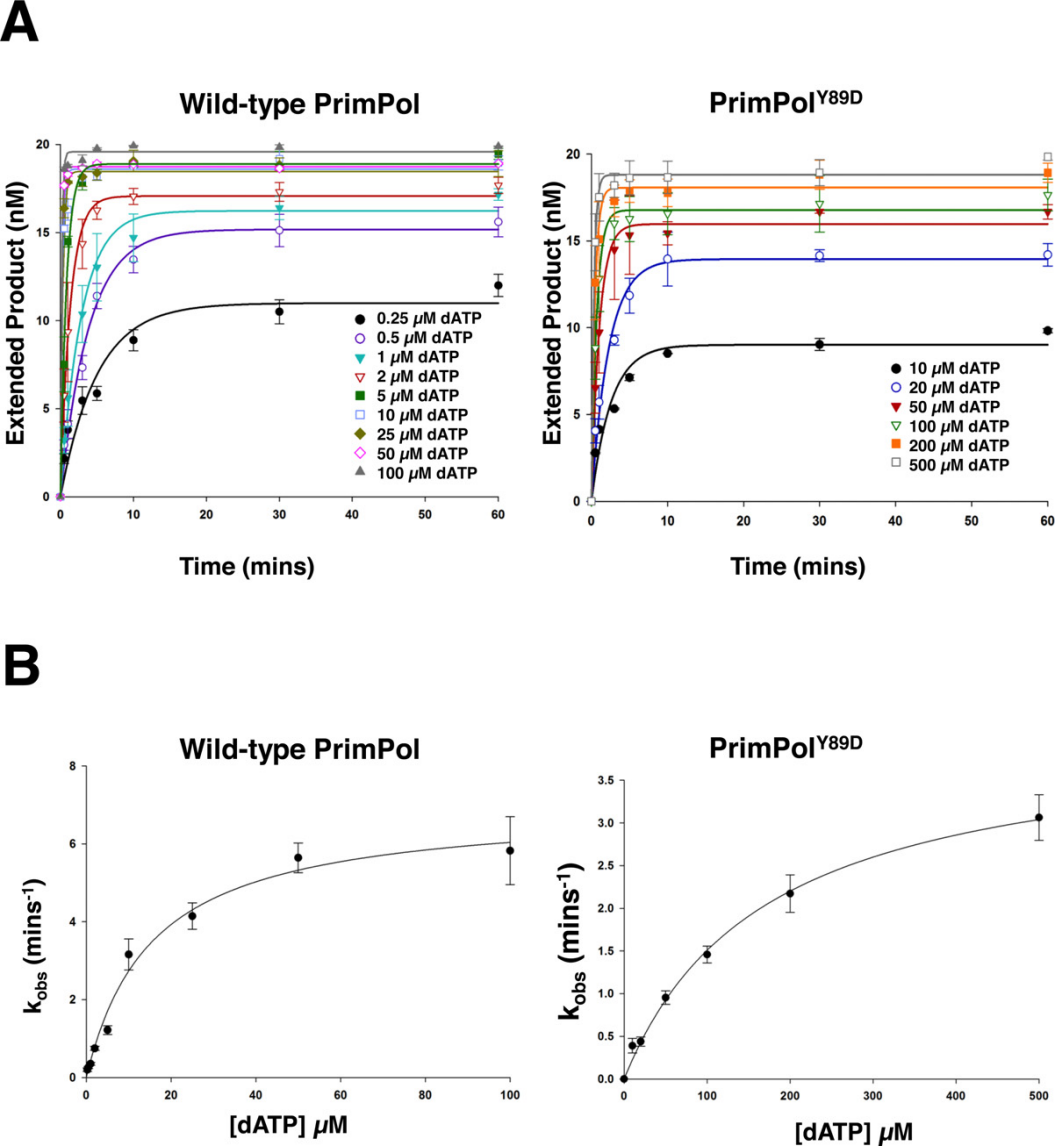
Kinetic analysis of human PrimPol and PrimPol^Y89D^. Single turnover kinetics of wild-type and PrimPol^Y89D^ to determine catalytic efficiency (*k*_pol_) and dNTP binding efficiency (*K*_D(dNTP)_). (A) The concentration of extended DNA product, as determined by electrophoresis, was plotted against time and fit to an exponential curve for a number of dATP concentrations for wild-type and PrimPol^Y89D^. These data were fit to exponential curves as described in Equation (1) and *k*_obs_ was determined. (B) *k*_obs_ was subsequently plot against dATP concentration for wild-type PrimPol and PrimPol^Y89D^ and these data were fit to hyperbolic curves as described in Equation (2) to determine *k*_pol_ and *K*_D(dNTP)_. *k*_pol_ for wild-type PrimPol was found to be 6.98 min^−1^ ± 0.40 and *K*_D(dNTP)_ was 15.51 μM ± 2.71. *k*_pol_ for PrimPol^Y89D^ was 4.07 min^−1^ ± 0.19 and *K*_D(dNTP)_ was 170.21 μM ± 18.80.

To evaluate the effects of this mutation on the enzymatic properties of PrimPol, a kinetic analysis was performed to determine the catalytic efficiency (*k*_pol_) and binding constants for dATP (*K*_D(dNTP)_) opposite a templating thymine (Supplementary Figure S1). We found the wild-type *k*_pol_ to be 6.98 min^−1^ and the *K*_D(dNTP)_ to be 15.51 μM (Figure 6). PrimPol is an extremely slow enzyme—for comparison the *k*_pol_ of base excision repair enzyme DNA polymerase β is 1944 min^−1^ (17). We determined the Y89D variant of PrimPol to be a slightly slower polymerase, with a *k*_pol_ of 4.07 min^−1^ and binds dNTPs over 10-fold less competently, with a *K*_D(dNTP)_ of 170.21 μM (Figure 6). The reduction in catalytic efficiency and dramatic reduction in dNTP binding may or may not be dependent on the initial binding of DNA.

If dNTP coordination had been severely affected in the Y89D variant, we would expect quite a deviation from wild-type PrimPol in the fidelity of nucleotide incorporation. However, we found that the Y89D variant of PrimPol did not have any appreciable variation from the wild-type enzyme in terms of fidelity (Supplementary Figure S2). If there was an alteration in the coordination of incoming dNTPs, there would also be a lower probability that this enzyme would be able to bypass the highly distortive lesions that the wild-type enzyme could bypass. However, we observed that PrimPol^Y89D^ was able to synthesize through even highly distortive lesions, such as a 6–4 photoproduct (Supplementary Figure S3).

Aromatic residues often act as a steric gate for sugar discrimination (18). Mutation of these key residues can result in the incorporation of rNTPs instead of dNTPs opposite a templating DNA strand (19). PrimPol^Y89D^, like wild-type PrimPol, was also unable to extend primers using rNTPs (Supplementary Figure S4). Therefore, it is highly unlikely that this tyrosine residue is involved in discerning the sugar of the incoming nucleotide, particularly as the Y89D variant also loses its ability to prime using rNTPs.

### Y89D mutation induces significant structural changes to PrimPol

**Figure 7.**
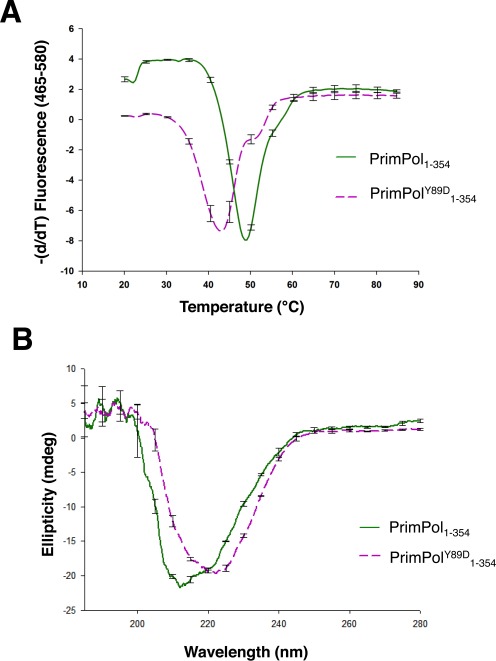
Y89A induces a structural perturbation in PrimPol. The polymerase domain of the Y89D variant of PrimPol has a different global structure and thermal stability compared to wild-type PrimPol. (A) Thermal denaturation of the polymerase domain of PrimPol and its Y89D variant. The negative differential of the fluorescence profiles of the polymerase domains (PrimPol_1–354_) of wild-type PrimPol (solid green line) and its Y89D (broken purple line) variant plotted against temperature to determine their melting points. The melting point of the polymerase domain of the wild-type enzyme was found to be 49.12°C, while the Y89D variant had a considerably lower melting point at 42.50°C. (B) CD spectra of the polymerase domains of wild-type PrimPol and PrimPol^Y89D^ show a substantial difference in the global structure of PrimPol^Y89D^ relative to the wild-type enzyme.

The observed reduced enzyme activity of PrimPol^Y89D^ could be the result of the loss of aromatic interactions involved in local base stacking events with either the template or the incoming nucleotide. However, it may instead be required for maintaining the structural integrity of the enzyme and mutation of this tyrosine causes more global effects in the structure of PrimPol. To test this second possibility, we subjected the polymerase domains of both the wild-type and PrimPol^Y89D^ to thermal denaturation and CD to check for alterations. There was a striking difference in both the thermal denaturation profiles and the CD spectra of the two enzymes (Figure 7), suggesting this point mutant results in global changes to the enzyme that causes the reduction in activity observed above. The melting point of the polymerase dropped from 49.12°C for the wild-type polymerase domain compared to 42.50°C for PrimPol^Y89D^ (Figure 7A). This significant drop in melting temperature suggests that the enzyme has become less stable and more structurally disordered following the point mutation. Analyses of the secondary structure from the CD spectra of the two variants’ polymerase domains by the variable selection algorithm (CDSSTR) (20), which provides better fits for globular proteins, revealed a difference in the α-helical content of these domains (37% for wild-type PrimPol compared with 21% for Y89D), as well as β-strand content (25% for wild-type compared with 29% for Y89D). Additionally, this analysis predicted 30% of the structure of the PrimPol^Y89D^ polymerase domain to be unstructured, whereas just 24% of wild-type was predicted to be unstructured.

### PrimPol^Y89D^ significantly decreases DNA replication fork rates *in vivo*

**Figure 8.**
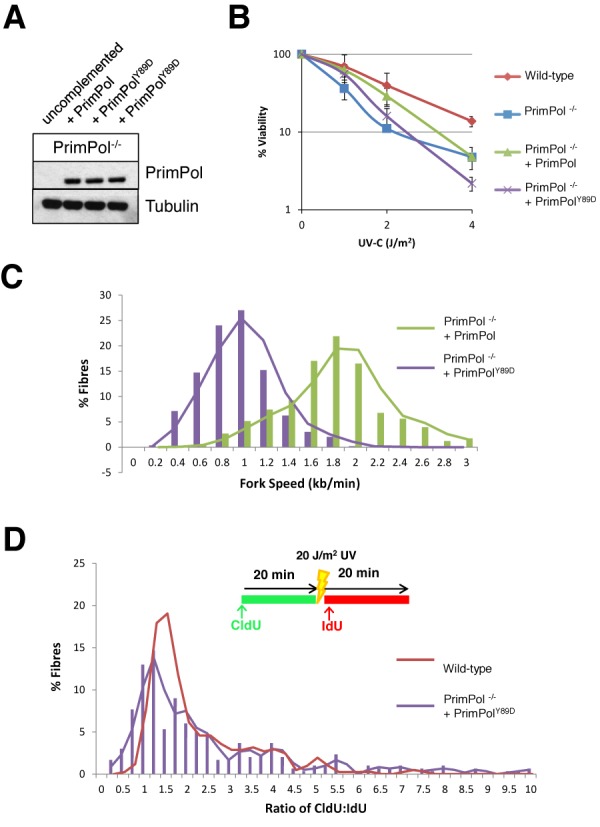
PrimPol Y89D cannot fully complement the UV sensitivity seen in PrimPol^−/−^ DT40 cells due to decreased replication fork speeds. (A) Western blot showing overexpression of wild type and Y89D point mutant forms of PrimPol in PrimPol knockout (PrimPol^−/−^) DT40 chicken cells. (B) Cell viability was measured using CellTiter-Blue 48 h after increasing doses of UV-C were administered to the different cell types. Results represent three independent repeats and the error bars represent the standard deviation across the repeats. (C) DNA fibres were measured after sequential labelling with CldU and IdU for 20 min each to calculate replication speed. Results represent three independent repeats. (D) A 20 J/m^2^ UV-C pulse was included between the two labels and the ratio of the pre- and post-UV-C labels was calculated, with increased ratios representing replication stalling after UV. Results represent three independent repeats.

To gain further insights into how the Y89D mutant form of PrimPol may contribute to the myopic phenotype observed in human patients, we studied the influence of PrimPol^Y89D^ on DNA replication in damaged and undamaged cells. We utilized a DT40 PrimPol deleted cell line (PrimPol ^−/−^) (4) to examine the ability of PrimPol^Y89D^ to complement this loss, as previously reported for other mutant forms of PrimPol (14). We stably expressed human PrimPol^Y89D^ at similar levels to WT PrimPol in PrimPol knockout cells (Figure 8A), with no obvious effects on cell growth.

We reported previously that PrimPol^−/−^ cells have a greater sensitivity to UV irradiation thus we first examined the effects of increasing doses of UV-C on PrimPol^Y89D^ complemented cell lines. Although PrimPol^−/−^ cells complemented with wild-type PrimPol showed a reversal of this sensitivity, cells complemented with PrimPol^Y89D^ maintained a decreased viability after UV damage (Figure 8B). Next, we measured the replication fork speeds in PrimPol complemented cell lines using DNA fibre analysis, as previously described (4,14). Strikingly, we observed a dramatic decrease in fork speeds in cells complemented with PrimPol^Y89D^ (0.84 kb/min) compared with cells carrying the wild-type PrimPol (1.66 kb/min) (Figure 8C). This decrease in fork speed was significantly greater than that previously reported in PrimPol^−/−^ cells (1.45 kb/min; (4,14)), suggesting the Y89D mutant form of PrimPol may actually impede on-going DNA replication. In contrast, PrimPol^Y89D^ complemented the increased stalling defect observed in PrimPol^−/−^ cells treated with UV-C, equally as well as WT PrimPol (Figure 8D).

## DISCUSSION

A recent exome sequencing study has implicated a point mutation (Y89D) in the DNA damage tolerance polymerase, PrimPol, in high myopia in human patients. However, this report did not investigate the molecular consequences of this genetic alteration. Here, we report that the Y89D variant of PrimPol, previously found to be associated with high myopia, is a low processivity variant of PrimPol. Unlike wild-type PrimPol, it inserts just one nucleotide in a single binding event and has a significantly lower affinity for both dNTPs and DNA. Notably, this mutant did not exhibit any alteration in the fidelity or TLS spectrum relative to wild-type PrimPol. To determine whether this altered activity was a result of losing the hydrophilic hydroxyl moiety or the hydrophobic ring moiety, we constructed two more variants in which tyrosine 89 was mutated to either phenylalanine or serine. The Y89F mutant restored processivity to near-wild-type levels but Y89S had a similar activity to Y89D. In addition, we found that the Y89D PrimPol variant was unable to produce ribonucleotide primers, but this activity was restored in the Y89F variant, indicating that PrimPol requires a hydrophobic aromatic ring at position 89 to maintain processivity but the presence of a hydroxyl moiety is dispensable.

Insights from structural studies on primases and polymerases suggest two different potential explanations for the reduction in polymerase processivity observed in this study. The conserved tyrosine at position 89 could have an important role in base stacking or could be required for the structural integrity of the protein. There is an invariant phenylalanine in nonhomologous end-joining (NHEJ) AEPs that plays a vital role in the recognition and orientation of both the templating and the incoming bases through stacking interactions with both bases and is responsible for moving the incoming nucleotide into the enzyme's active site (21,22). Phenylalanine 290 in human Pol η helps form the hydrophobic core of the thumb domain (23). Mutation of this residue is associated with the genetic disorder *xeroderma pigmentosum variant*, which is characterized by a susceptibility to skin cancers caused by a failure to perform proficient TLS opposite UV lesions. This mutation negates polymerase activity and is predicted to destabilize the thumb domain, resulting in reduced primer binding. While the structure of PrimPol has not yet been elucidated, it is conceivable that the tyrosine at position 89 is involved in preserving the structure of a domain analogous to the thumb domain that is found in other polymerases involved in binding/coordinating the templating DNA strand. Tyrosine 89 is situated in a conserved hydrophobic pocket containing other conserved aromatic residues including Y87, F88 and Y90, as well as a hydrophobic residue at position 86 (Figure 1) and it is likely that disruption of Y89 disrupts a conserved interaction between these amino acids. A secondary structure prediction from PSIPRED (24) predicts that all of these amino acids lie within an α-helical region and the Y89 mutation may disrupt this helix. The CD data certainly supports this model, indicating that the Y89D variant has less α-helical structure than the wild-type. We postulate that the Y89D mutation causes a disruption in the global structure of the polymerase domain of PrimPol. As the affinity for DNA and dNTPs is decreased in the Y89D variant, but the apparent fidelity of the enzyme remains unchanged, we suggest that dNTP binding is dependent on DNA binding, which is disrupted by the structural changes induced by this mutation.

*In vivo* analysis identified a dramatic decrease in replication fork rates in cells expressing PrimPol^Y89D^ and, despite being able to complement fork stalling in PrimPol^−/−^ cells after UV damage, it was unable to prevent decreased cell viability. Consistent with *in vitro* assays, PrimPol^Y89D^ causes decreased replication fork rates in cells and it appears that this defect is enough to decrease the damage tolerance of these cells. Both our *in vivo* and *in vitro* studies on this mutant indicate that although PrimPol^Y89D^ retains the ability to bypass lesions, its capacity to replicate DNA is severely compromised. Therefore, it appears that although PrimPol^Y89D^ is recruited to stalled replisomes, the underlying replication defect causes a significant slowing of replication forks, potentially resulting in incomplete lesion bypass, causing cells to progress through the cell cycle with underreplicated genomic DNA. Clearly, these cells carry an abundance of PrimPol compared to wild-type levels and thus replication fork speed alone in endogenous mutants may not be so significantly altered. However, in cases where PrimPol is actively recruited to on-going forks or damage sites, this ‘slow’ mutant is likely to cause significant problems. Therefore, unexpectedly, it appears that harbouring the PrimPol^Y89D^ mutation is more detrimental than not having PrimPol at all. This data correlates with the phenotypes observed in patients carrying this mutation as problems are observed in tissues where levels of UV damage are expected to be high. Thus, in tissues where PrimPol is recruited in higher abundance to replicate through DNA damage, such as eyes, cellular defects are much more likely to be prevalent.

The link between reduced PrimPol polymerase activity and myopigenicity remains unclear. Previous studies into the genes that predispose high myopia have identified two clusters of mutations, one in the outer retina affecting photoreceptors and ON-bipolar cell function and another in the scleral extracellular matrix composition and metabolism (11). Significantly, mutations in PrimPol do not fit into either category. The severity of the clinical phenotype of patients with the Y89D mutation varied (9) and it is highly likely that this gene is not a singular, unique aetiological factor and that other genetic and environmental factors also contribute to pathogenesis. Although replication defects associated with PrimPol^Y89D^ suggests that it contributes to the reported clinical phenotypes, further work is required to establish if a direct link exists between replication stress and onset of high myopia.

## Supplementary Data

Supplementary Data are available at NAR Online.

SUPPLEMENTARY DATA
