# Supplementary material for: Human PrimPol mutation associated with high myopia has a DNA replication defect
Source: Nucleic Acids Res. 2014 Sep 27;42(19):12102–11. doi: 10.1093/nar/gku879 (PMC4231748; doi:10.1093/nar/gku879)
Supplement: SUPPLEMENTARY DATA [file supp_42_19_12102__index.html]

Human PrimPol mutation associated with high myopia has a DNA replication defect — Human PrimPol mutation associated with high myopia has a DNA replication defect — SUPPLEMENTARY DATA 

# Human PrimPol mutation associated with high myopia has a DNA replication defect

## SUPPLEMENTARY DATA

**Files in this Data Supplement:**

- SUPPLEMENTARY DATA
