# Supplementary material for: Human PrimPol mutation associated with high myopia has a DNA replication defect
Source: Nucleic Acids Res. 2014 Sep 27;42(19):12102–11. doi: 10.1093/nar/gku879 (PMC4231748; doi:10.1093/nar/gku879)
Supplement: SUPPLEMENTARY DATA [file supp_gku879_nar-01669-h-2014-File010.pdf]

Table S1

#	**Primer**	**Sequence**
1	PrimPol^{Y89D} Forward Primer	5'-GAATTTTGGTTTGACTATAAATCCAGAAAAAATCTCTTACACTGCTATG-3'
2	PrimPol^{Y89D} Reverse Primer	5'-GATTTATAGTCAAACCAAAATTCAGCATAGGTTGTCACAAG-3'
3	PrimPol^{Y89S} Forward Primer	5′-GAATTTTGGTTTTCATATAAATCCAGAAAAAATCTCTTACACTGCTATG-3′
4	PrimPol^{Y89S} Reverse Primer	5′-GATTTATATGAAAACCAAAATTCAGCATAGGTTGTCACAAG-3′
5	PrimPol^{Y89F} Forward Primer	5′-GAATTTTGGTTTTTCTATAAATCCAGAAAAAATCTCTTACACTGCTATG-3′
6	PrimPol^{Y89F} Forward Primer	5′-GATTTATAGAAAAACCAAAATTCAGCATAGGTTGTCACAAG-3′

Supplementary Table 1: Primers used in site-directed mutagenesis of PrimPol and PrimPol$_{1\text{-}354}$ to produce the PrimPol^{Y89D}, PrimPol^{Y89S} and PrimPol^{Y89F} variants.

Table S2

#	Oligonucleotide	Label	Sequence
1	Poly(dA)$_{60}$	5'-Biotin	5'-AAAAAAAAAAAAAAAAAAAAAAAAAAAAAAAAAAAAAAAAAAAAAAAAAAAAAAAAAAAA-3'
2	Poly(dC)$_{60}$	5'-Biotin	5'-CCCCCCCCCCCCCCCCCCCCCCCCCCCCCCCCCCCCCCCCCCCCCCCCCCCCCCCCCCCC-3'
3	Poly(dG)$_{60}$	5'-Biotin	5'-GGGGGGGGGGGGGGGGGGGGGGGGGGGGGGGGGGGGGGGGGGGGGGGGGGGGGGGGGGGG-3'
4	Poly(dT)$_{60}$	5'-Biotin	5'-TTTTTTTTTTTTTTTTTTTTTTTTTTTTTTTTTTTTTTTTTTTTTTTTTTTTTTTTTTTT-3'
5	HP-20 Primer	5'-Hex	5'-TGTCGTCTGTTCGGTCGTTC-3'
6	ND-50 Template$_{TT}$	None	5'-CGCGCAGGGCGCACAACAGCCTTGAAGACCGAACGACCGAACAGACGACA-3'
7	HP-27 Primer	5'-Hex	5'-TGTCGTCTGTTCGGTCGTTCGGTCTTC-3'
8	ND-50 Template$_{AA}$	None	5'-CGCGCAGGGCGCACAACAGCCAAGAAGACCGAACGACCGAACAGACGACA-3'
9	ND-50 Template$_{CC}$	None	5'-CGCGCAGGGCGCACAACAGCCCCGAAGACCGAACGACCGAACAGACGACA-3'
10	ND-50 Template$_{GG}$	None	5'-CGCGCAGGGCGCACAACAGCCGGGAAGACCGAACGACCGAACAGACGACA-3'
11	HP-28 Primer	5'-Hex	5'-TGTCGTCTGTTCGGTCGTTCGGTCTTCA-3'
12	6-4(PP) Template	None	5'- CTCGTCAGCATCT^TCATCATACAGTCAGTG-3'
13	HP-16 Primer	5'-Hex	5'-CACTGACTGTATGATG-3'
14	CPD Template	None	5'-CGCGCAGGGCGCACAACAGCCT=TGAAGACCGAACGACCGAACAGACGACA-3'
15	8-oxo-G Template	None	5'-CGCGCAGGGCGCACAACAGCC8-oxo-GTGAAGACCGAACGACCGAACAGACGACA-3'
16	dUracil Template	None	5'-CGCGCAGGGCGCACAACAGCCUTGAAGACCGAACGACCGAACAGACGACA-3'
17	AP Template	None	5'-CGCGCAGGGCGCACAACAGCCAPTGAAGACCGAACGACCGAACAGACGACA-3'
18	TG Template	None	5'-CGCGCAGGGCGCACAACAGCCTGTGAAGACCGAACGACCGAACAGACGACA-3'

Supplementary Table 2: Sequences of the DNA oligonucleotides used in biochemical assays. For each substrate the labels, if any, are given. Any lesions in the DNA are denoted in red in their respective sequences.

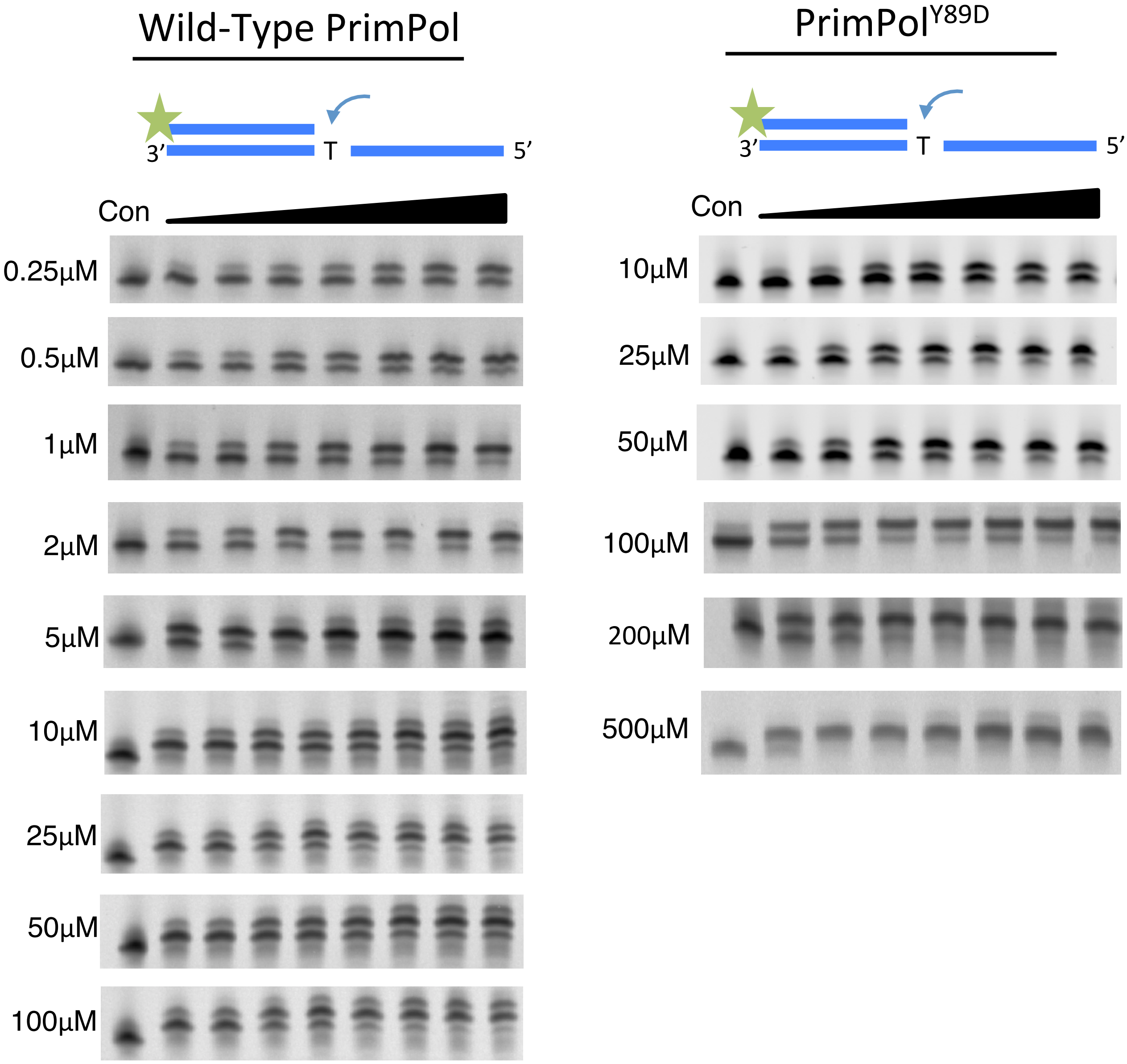

Supplementary Figure 1: Representative single incorporation extension assays for each of the concentrations of dATP tested (See graphs in Figure 6) showing extension relative to the control bands in the first lane. Each of these gels were repeated in triplicate.

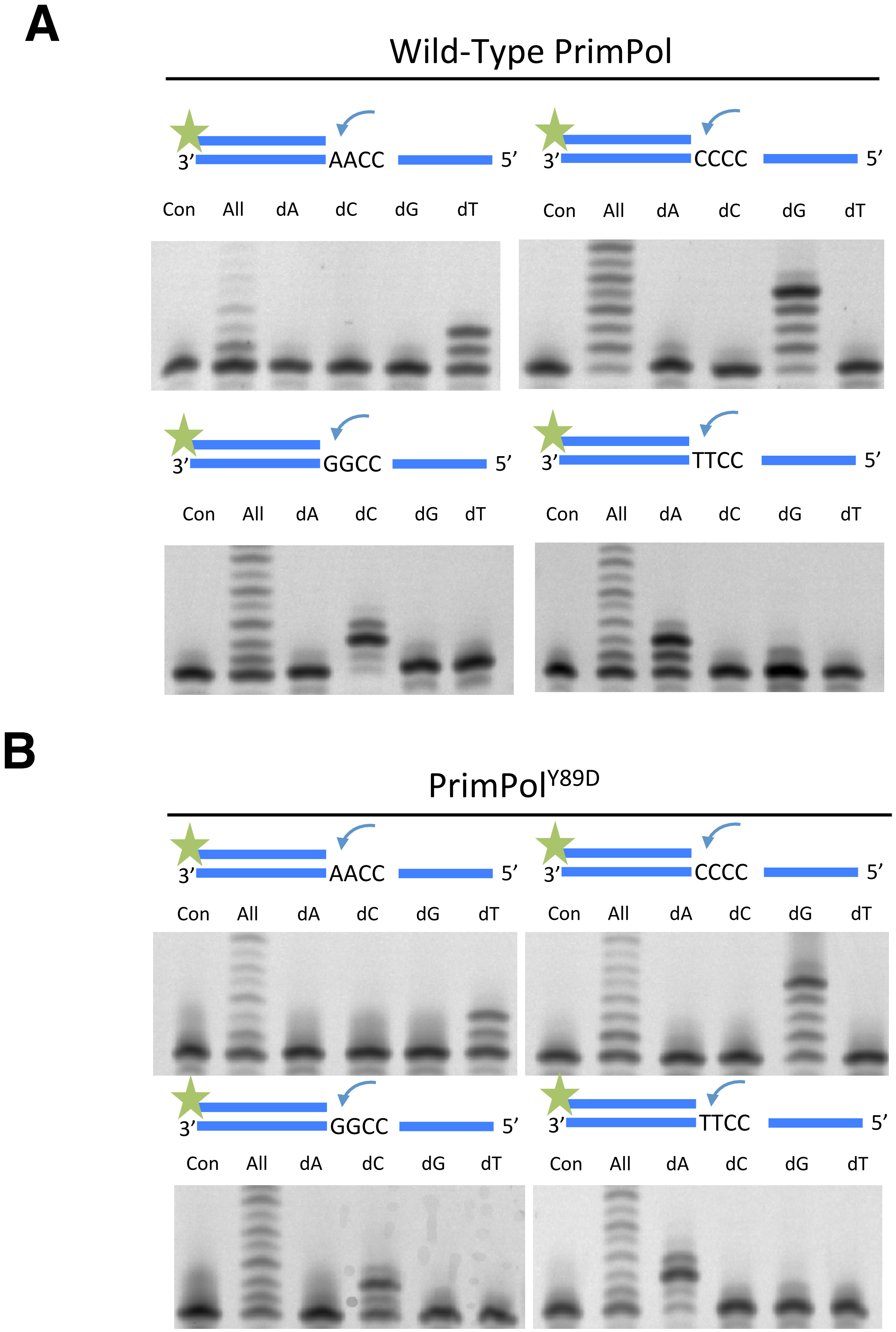

Supplementary Figure 2: The fidelity of the PrimPol^{Y89D} variant remains unchanged relative to the wild-type PrimPol. **(A)** Wild type PrimPol was incubated for 5 minutes in the presence of no dNTPs, all dNTPs and each of the four individual dNTPs opposite different templating bases. **(B)** To provide comparable activity between the two constructs, the PrimPol^{Y89D} construct was incubated for 30 minutes.

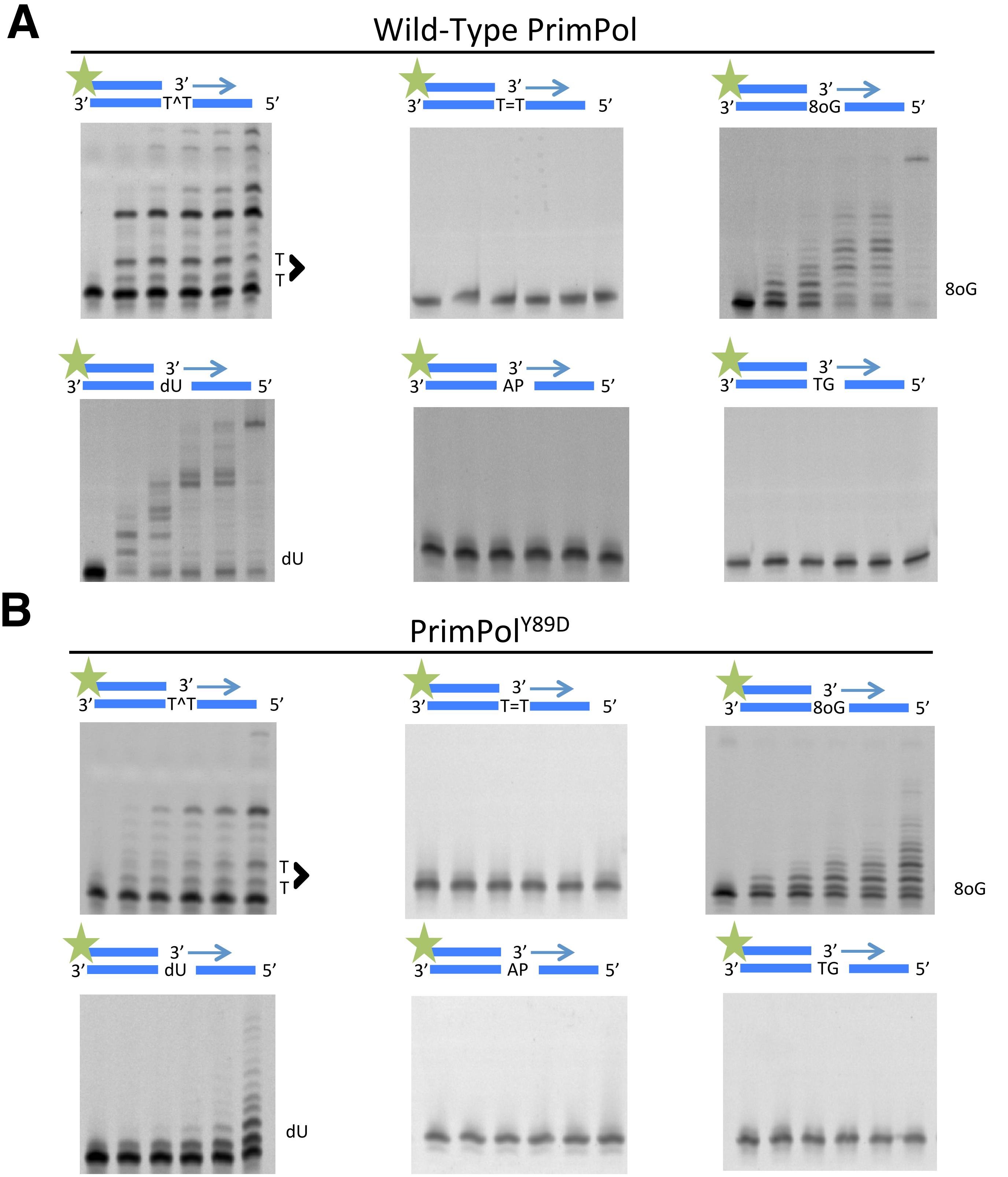

Supplementary Figure 3: The translesion DNA synthesis spectrum of the PrimPol^{Y89D} variant remains unchanged. **(A)** PrimPol has the ability to bypass a 6-4 photoproduct (top-left), an 8-oxoguanine moiety (top-right) and deoxyuracil (bottom-left). Wild-type PrimPol cannot read-through a CPD (top-center), apurinic/apyrimidinic site (bottom-center) or a thymine glycol lesion (bottom-right). **(B)** PrimPol^{Y89D} retains its TLS activity as the wild-type protein, albeit with lower polymerase activity. In each case, the first lane represents a control and lanes 2-6 represent incubation periods of 0.5, 1, 3, 5 and 60 minutes respectively.

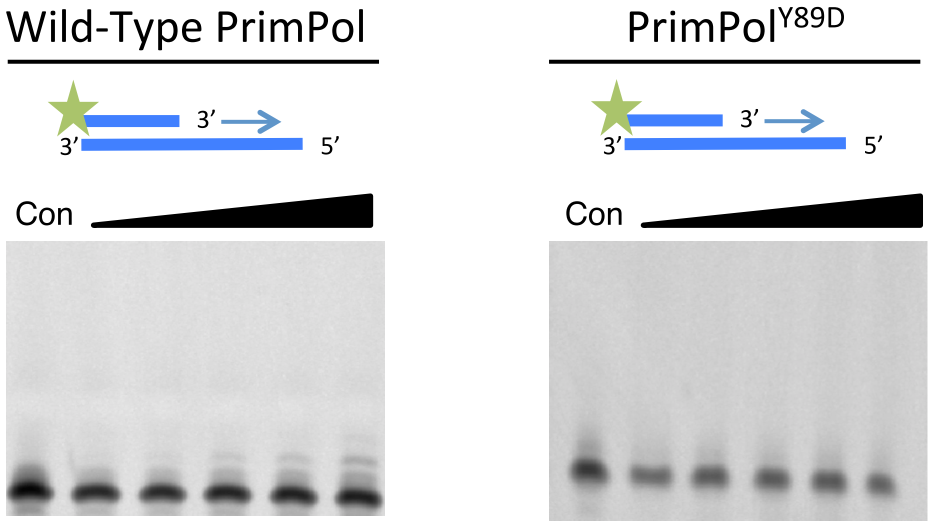

Supplementary Figure 4: Neither wild-type PrimPol, nor PrimPol^{Y89D} is able to extend DNA primers using ribonucleotides efficiently. In each case, the first lane represents a control and lanes 2-6 represent incubation periods of 0.5, 1, 3, 5 and 60 minutes respectively.
